# Exploratory survey study of long-term users of nicotine replacement therapy in Danish consumers

**DOI:** 10.1186/1477-7517-12-2

**Published:** 2015-01-19

**Authors:** Gitte Borup, Kim Lyngby Mikkelsen, Philip Tønnesen, Lona Louring Christrup

**Affiliations:** Department of Drug Design and Pharmacology, University of Copenhagen, Universitetsparken 2, 2100 Copenhagen, Denmark; The Danish Patient Compensation Association, Copenhagen, Denmark; Danish Centre for Sleep Medicine, Glostrup Hospital, Glostrup, Denmark

**Keywords:** Nicotine replacement therapy, NRT, Long-term use, Nicotine dependence, Modified Heaviness of Smoking Index, Survey research, User perspective

## Abstract

**Background:**

Long-term use of nicotine replacement therapy (NRT) has been approved in several countries for smokers who are unable or unwilling to quit smoking. However, information on basic characteristics, degree of nicotine dependence, health status and contentment with long-term use of NRT is scarce. The aim of this study was to collect information on the characteristics of long-term NRT users, having used NRT for at least 12 months, reasons for, and contentment with, their continued use of NRT including reasons for wishing to quit or sustain use and an estimation of their degree of nicotine dependence.

**Method:**

Through advertisements in three national Danish newspapers, long-term NRT users were recruited to answer a short questionnaire about basic characteristics, health status and satisfaction with using NRT. A modified version of the Heaviness of Smoking Index (HSI) questionnaire was applied to estimate nicotine dependence. Linear regression was used to test association between time to first NRT and daily dosage of NRT.

**Results:**

A total of 92 respondents were included in the data analysis. A majority of 88% wished to quit NRT for the following reasons: costs of NRT, being tired of feeling addicted and fear of adverse health effects. Scoring on the modified HSI scale was 22.0% low, 68.0% moderate and 9.3% high dependent. Of the respondents, 67.0% used NRT within the first 30 min after waking. A validation check found a significant linear association between the two items in the modified HSI.

**Conclusion:**

A significant majority of users wished to quit NRT because of the cost of products, being tired of feeling addicted and fear of adverse health consequences. The majority of these users were moderate to high nicotine dependent. The strong association found between time to first NRT and NRT dosages used per day gives reason to believe the validity of the modified HSI. Further studies are required for confirmation. Better counselling of long-term users on the benefits of using NRT compared to smoking should be provided, for those who are chronically dependent, as well as support to stop long-term use of NRT if wanted.

## Background

Results from a national survey on smoking habits, conducted in 2012 on a representative segment of adults in Denmark (DK), indicated that among respondents using nicotine replacement therapy (NRT), 48% (66 out of 138) had used the products for more than 12 months [1] being equivalent to 1.3% of the total adult population in DK. When NRT first became available, the recommended duration of treatment was up to 3 months after smoking cessation for most products, since this was the duration of use in most clinical trials designed to determine efficacy and safety of NRTs [2]. The indications of most NRT products have since been extended to include concurrent use while smoking and allow for longer durations of treatment, as it was recognised that this would benefit the more dependent smokers in succeeding in quitting [3]. In the literature, the definition of long-term use of NRT ranges from 3 to 12 months [4–7]. In this study, long-term use is defined as the daily use of NRT for 12 months or more, which is the current longest recommended duration of use, in the summary of products characteristics (SPC) for nicotine gum in DK.

Reports on the incidence of becoming a long-term user of NRT following smoking cessation attempts vary from 1% in real life settings [8] up to 25% of the successful abstainers participating in clinical trials on smoking cessation [9–12]. Generally, smokers enrolled in smoking cessation studies have higher levels of nicotine dependence than the average smokers in the general population and thus have a greater likelihood of becoming long-term users of NRT [5–7, 10].

Initially, long-term use of NRT was viewed as problematic because of concerns about possible adverse health consequences caused by nicotine [13, 14]. Some research has indicated that NRT can worsen insulin resistance and prevent the normalisation of cholesterol levels, otherwise obtained following smoking cessation [15–17], but to this date, no serious adverse effects have been reported. Also, no causality between acute cardiovascular episodes in high-risk populations and the use of NRT has been found [18]. In fact, long-term use of NRT and other non-combustible tobacco products are advocated for as a way of harm reduction for smokers who are incapable or unwilling to quit smoking [5, 8, 19–22]. Current smoker precautions to using NRT long-term include cost, fearing becoming addicted to NRT and fearing potential adverse health effects [23]. However, results from previous research have shown that smokers are willing to use NRT with the goal of either reducing or quitting smoking, or as a possible long-term substitute for cigarettes, when the health advantages compared to smoking have been explained to them [22, 24–29]. Results from an internet survey of nicotine gum users, where long-term use was defined as gum use for more than 3 months, suggested that users scoring high on modified nicotine dependence scales were less likely to have quit using NRT at follow-up 1 month later and that 75.6% of the long-term users felt unable to quit using nicotine gum [4].

The purpose of the present study was therefore to collect information on the characteristics of long-term NRT users, that had used NRT for more than 12 months, reasons for their continued use of NRT and contentment with being long-term users of NRT, including reasons for wishing to quit or sustain use. Furthermore, the collected data gave us the opportunity to estimate the degree of nicotine dependence in the long-term users and make a validation check of a modified version of the Heaviness of Smoking Index (HSI) scale.

## Methods

### Recruitment

Advertisements were posted in three national newspapers for 1 day, seeking daily users of NRT to participate in the survey. Respondents should either be former or current smokers, have used NRT for more than 12 months and be at least 18 years of age. Simultaneously with the printed ad, an online banner was run for 14 days on two of the newspapers’ online versions from where the respondents could complete the questionnaire directly. Participation gave an opportunity to win one of five gift vouchers of 500 DKK (70 Euros). The survey was run in two parts, the first two advertisements in November 2012 and the second in January 2013. The investigation was approved by the Danish Data Protection Agency. The participants where informed that they could discontinue their participation at any point, and have their answers withdrawn from the investigation. Only answers from completed questionnaires were included in the results.

### Questionnaire and ratings

The questionnaire covered basic demographics, current use of NRT products, reasons for using NRT, reason for wishing to quit or sustain NRT use, present and former smoking history, whether or not respondents had been encouraged to quit smoking, received advice on correct use of NRT, health status and contact information if the respondents wished to participate in a future interview investigation, regarding their experience as long-term NRT users. In the second round, one question was added to the survey, asking former smokers if they believed that they would relapse to smoking if NRT where no longer available [30]. Although e-cigarettes are not medicinal products, it was possible for the respondents to choose e-cigarettes when asked about their NRT products of choice. The argument for including e-cigarettes was a presumption that the general population, to some extent, does not differentiate between NRT and e-cigarettes as aids for quitting smoking. E-cigarettes were not mentioned in the wording of the advertisement, but when filling in the questionnaire, respondents had the opportunity to select e-cigarette as their nicotine source, leaving it to the respondent to define e-cigarettes as NRT or not.

To evaluate degree of nicotine dependence, a modified version of the HSI scale was used, where pieces of acute-acting single-dose NRT was substituted for cigarettes in a 1:1 fashion. The HSI is a two-item questionnaire [31] that is based on the Fagerström test for Nicotine Dependence (FTND) [32]. The outcome of HSI, in association with smoking, has been found to correlate highly with biochemical dependence measures [33] and to be a strong predictor of abstinence outcomes as well as predictors of tendency to relapse [31, 34]. The HSI comprises a question about “time to first cigarette in the morning” that has been found to be a reliable single measure of nicotine dependence [34], which was therefore analysed independently. The second question was “use of pieces of NRT per day”. The categorization of NRT use was done subsequently. The modified HSI score was calculated as the sum score of the two items, and the levels of nicotine dependence were categorised as low (0–1 points), moderate (2–4 points) and high (5–6 points) [35].

### Statistics

To undertake a validation check of the HSI scale, a linear regression was used in order to assert a negative association and to measure the strength of association between time to first NRT (TTFN) and NRT use (pieces/day) and between TTFN and recalled smoking (cig/day). TTFN was categorised in four groups and used as an explanatory variable, either as a categorical variable with group A as the reference group or as a continuous variable. The likelihood ratio test was used to test for linear trend, comparing models with TTNF used as a categorical variable and with TTNF used as a continuous variable. Linear regression was also used to find the equivalence ratio between daily NRT use (NRT pieces/day) and recalled smoking (cig/day). Fractional polynomial regression was used to give a graphical check of the association. As there was clearly a non-linear association, the analysis was done separately for individuals with a smoking history less or equal to 25 cig/day and for individuals with a smoking history of more than 25 cig/day. In this analysis, the delta-beta influence statistics was used to identify outliers. Delta-beta is the difference between the regression coefficient when the *j* th observation is included and excluded. Observations were excluded if delta-beta was >5 standard deviation. A significance level of 0.05 was used in all tests. All statistical analyses were done using Stata 13.1 (StataCorp, 4905 Lakeway Drive, College Station, TX 77845 USA).

## Results

A total of 112 respondents completed the questionnaire, 95 former smokers, 17 current smokers and 1 never smoker. Data from current smokers was not included in the data analyses. Data from additionally 3 respondents were excluded from the analyses: 1 never smoker, 1 who had quit using NRT 3 years previously and 1 being a former pipe smoker.

### Characteristics of respondents

Respondent characteristics are listed in Table 1. The most common NRT product used by the participants’ was nicotine chewing gum 2 mg (67.4%, *N* = 62), followed by 4 mg gum (12.0%, *N* = 11) and inhaler (9.8%, *N* = 9). Other products used were lozenges, e-cigarettes and chewing tobacco. Thirteen respondents combined two or more products. Four of the respondents (4.3%) used nicotine patches in combination with an acute-acting NRT. Some respondents stated their former daily cigarette use in intervals, e.g. 15–20. In these cases, the average of the interval given was used in the data analysis.

**Table 1:** **Basic characteristics, smoking history and current NRT use**

Total of respondents (***n*** = 92)	
Men (%, *N*)	45.7 (42)
Mean age (SD, range)	52.0 (10.6, 33–75)
Mean duration of NRT use in years (SD, range)	5.5 (4.5, 1–27)
Mean current NRT use^ab^ (SD, range)	15.0 (9.4, 3–60)
Mean former smoking years (SD, range)	27.4 (11.5, 5–53)
Mean recalled smoking^c^ (SD, range)	19.5 (8. 38, 5–60)
Wish to quit NRT (%, *N*)	88.0 (81)
Felt addicted to NRT (%, *N*)	77.2 (71)

^a^Including respondents using only acute single-dose NRT.

^b^Expressed as NRT pieces/day.

^c^Expressed as cig/day.

When asked to elaborate in their own words why they wished to quit using NRT, the primary causes stated were 1) being tired of feeling addicted to NRT (51.1%, *N* = 45) 2) cost of NRT products (50.0%, *N* = 44) and 3) fear of adverse health effects (40.9%, *N* = 36). The respondents not wishing to quit (*N* = 12) stated that they believed NRT to be less hazardous to their health than cigarettes (*N* = 7), that NRT prevented them to relapse to smoking (*N* = 7) and that they enjoyed using NRT (*N* = 7). The average NRT use per day, based on those only using acute-acting single-dose NRT (*N* = 75), was for those not wishing to quit 11.3 NRT pieces/day (*N* = 8) and for those wishing to quit 15.5 NRT pieces per day (*N* = 67).

### Assessment of nicotine dependence

Nicotine dependence scores from the modified HSI were assessed only in respondents using acute-acting single-dose NRT (*N* = 75). The modified HSI was calculated from current NRT use and time to first NRT after waking in the morning (TTFN). It was found that 22.7% of the respondents were low nicotine dependent (*N* = 17), 68.0% were moderately dependent (*N* = 51) and 9.3% highly dependent (*N* = 7) (Table 2). The respondents who were highly dependent used a mean of 35.9 NRT pieces/day, the moderately dependent used an average of 14.3 NRT pieces/day and the low dependent used a mean of 8.7 NRT pieces/day.

**Table 2:** **Classification of dependence according to the Heaviness of Smoking Index (HSI score)**

	A = 3 points (0–5 min)	B = 2 points (6–30 min)	C = 1 point (31–60 min)	D = 0 points (later)
A = 0 points (≤10)	4	8	6^b^	8^b^
B = 1 point (11–20)	15	10	9	3^b^
C = 2 points (21–30)	4^a^	5	-	-
D = 3 points (31 ≤)	2^a^	1^a^	-	-

The classification of dependence includes respondents using only acute-acting *single-dose* NRT. Patch, e-cigarette and inhaler users were omitted (*n* = 75). The time intervals in the columns indicate time to first NRT after waking. The intervals given in the rows indicate number of NRT doses per day.

^a^Highly nicotine dependent respondents (score 5–6) = 9.3% (*N* = 7).

^b^Respondents found low dependent on nicotine (score 0–1) = 22.7% (*N* = 17). Respondents moderately dependent on nicotine (score 2–4) = 68.0% (*N* = 51).

Of all respondents using acute-acting NRT (*N* = 88) (patch users omitted *N* = 4), 67.0% (*N* = 49) stated a TTFN of 30 min or less. Again, of those using only acute-acting NRT (*N* = 75), 65.3% (*N* = 49) used NRT before 30 min after waking, using an average of 17.6 (SD = 10.3, range 5–60) NRT pieces/day. Those using NRT later during the day (N = 26) used an average of 10.2 (SD = 4.3, range 3–20) NRT pieces/day.

The strength and direction of the association between TTFN and amount of NRT pieces per day is shown in Table 3. The test for linear trend showed that the linear model was superior to the categorical model (likelihood ratio test: *p* = 0.23). For every category of delayed NRT use after waking, the average number of NRT pieces/day was reduced with 3.6 (95% CI −5.53 to −1.70) showing a highly significant association between TTFN and NRT use. Linear regression of recalled smoking with cig/day explained by TTFN (likelihood ratio test = 0.38) showed that cig/day was reduced by 2.77 per category of TTFN (95% CI −4.42 to −1.13).

**Table 3:** **Time to first NRT compared to mean current NRT use and recalled smoking**

Time to first NRT	% of respondents (N)	Mean NRT pieces/day (SD)	Mean recalled cig/day (SD)
0-5 min	33.3 (25)	19.1 (11.6)	22.2 (10.8)
6-30 min	32.0 (24)	16.1 (9.0)	20.4 (5.2)
31-60 min	20.0 (15)	11.2 (3.2)	17.4 (5.4)
Later	16.0 (11)	8.8 (5.5)	13.1 (5.1)

Includes respondents using only acute-acting *single-dose* NRT (*n* = 75). Respondents using patch, e-cigarette and inhaler were omitted. For every category of delayed NRT use after waking, the average number of NRT pieces/day was reduced to 3.6 (95% CI −5.53 to −1.70) showing a highly significant association between TTFN and NRT use.

### Equivalence ratio between current NRT pieces/day and recalled cig/day

**Figure 1:**
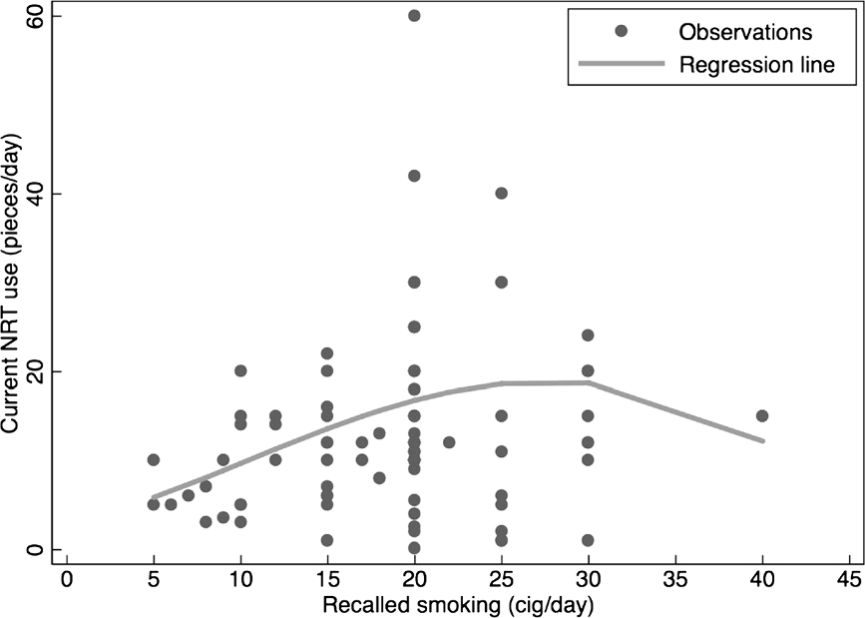
**Equivalence ratio between current NRT use and recalled smoking.** The regression line is based on a fractional polynomial regression and includes respondents using acute-acting single-dose NRT (*N* = 75). One outlier with high recalled cig/day and low NRT pieces/day was omitted (delta-beta = −8.1 SD). Logistic regression up to and including 25 cig/day showed a significant equivalence ratio of 0.86. No significance was found for those with recalled smoking of more than 25 cig/day.

The correlation between current NRT use and recalled smoking was low and insignificant (*r* = 0.18, *p* = 0.12) indicating that the association was also not linear for the full range of the data. Fractional polynomial regression showed that the association varied by the number of recalled cig/day, with a constant increasing equivalence ratio up to a maximum at approximately 25 cig/day and levelling off at higher recalled use. The linear regression of NRT use on recalled smoking (Figure 1) showed an equivalence ratio of 0.86 NRT/cig (95% CI 0.730 to 0.98, *N* = 64) for individuals with a smoking history of up to 25 (cig/day). No significant association for individuals with a smoking history of more than 25 (cig/day) (95% CI 11.9 to 20.1) could be demonstrated.

## Discussion

### Respondent characteristics

The characteristics of the respondents were found to be similar to the respondents in earlier studies on long-term NRT users [4, 5, 7] with regard to mean age, mean NRT use and duration of use, regardless that these studies had different inclusion criteria. Still, the generalizability of the characteristics found in this study cannot be validated. Long-term users of NRT who are concerned of or displeased with their NRT use may be more aware of news concerning NRT and thus more likely to participate in surveys on NRT resulting in an overrepresentation bias of this group, opposed to long-term users, who willingly continue using NRT [36].

### Dependence on nicotine in former smokers

When estimated from the modified HSI, including respondents using only acute-acting single-dose NRT (*N* = 75), 22% were found to rate low in nicotine dependence, 68.0% to rate moderate in dependence and 9.3% to rate high in dependence. Among Danish daily smokers, approximately 30% are estimated to be low dependent, 61% moderate dependent and 9% high dependent [37]. This might imply that smokers do not generally become more dependent on nicotine when switching to NRT than they were when still actively smoking. An explanation for the slightly lower proportion of low-dependent NRT users (22%), seen in this study compared to low-dependent smokers (30%) in the report by Glümer et al. [37], might be that low-dependent smokers succeed in quitting without becoming long-term users of NRT or that they quit smoking unaided.

The behavioural item of the HSI: how soon after waking smokers have their first cigarette (TTFN), has been suggested as a possible single-item measure of nicotine dependence, as time to first cigarette is highly negatively correlated to the full FTND scale [34]. When looking at TTFN, 67.4% of the respondents used NRT within the first 30 min after waking, almost equalling the moderate- and high-dependent users combined from the modified HSI. The negative and strong association between TTFN and NRT use found in the present study gives reason to believe the validity of the modified HSI, although extensive validation studies are needed for confirmation.

### Applicability of the heaviness of smoking index

When comparing smoking history in the following context, a caveat on bias in regard to the recalled smoking history must be emphasised. First, when asking respondents to recall habits exercised several years earlier, the accuracy of the recollected habits may be uncertain. Second, several respondents stated an interval of former average cigarette use, e.g. 15–20 cig/day from where an average was used.

However, assuming that the information on average cigarette consumption was accurate, regression analysis showed a reduction in NRT use (pieces/day) of 0.86:1 when compared to recalled smoking (cig/day). An upper limit of daily NRT use seemed to be present at 20 NRT pieces/day, with only eight respondents using more. The decreased use of NRT, as compared to the former daily cigarette use, has several plausible explanations. First, as earlier suggested by Cinciripini et al., the current NRT habits, may have replaced only the high-preference smoking moments, omitting the low-preference moments [38]. This underscores that the use of NRT at a lower rate compared to former smoking is not merely habitual, but instead driven by dependence. Second, when using nicotine gum, a physical limitation of jaw pain from constant chewing may also limit the daily consumption [39]. Third, the average recalled smoking history was on average 19.5 (cig/day) equalling the size of cigarette packets, and the average NRT use was 15.0 (pieces/day) equalling a blister pack of nicotine chewing gum. Some former smokers may therefore simply have adjusted their habits of one cigarette packet a day to one blister pack a day.

Also, no distinction was made between the strength of the acute-acting NRT products used by the respondents or the products different nicotine uptake profiles, when estimating nicotine dependence. This would have contributed to a more precise estimation of nicotine dependence, but it would also have demanded further explanations from the participants on how they use the products, and whether or not they used them correctly. For instance, some long-term users may purchase 4 mg chewing gum and use 10 pieces/day, but instead of using them as intended, they may in fact divide each piece into halves obtaining 20 2 mg pieces/day. Others use 30 2 mg chewing gums/day but may not let them rest in-between chewing, resulting in the nicotine being swallowed instead of absorbed through the oral mucosa. Being able to use NRT in places where smoking was prohibited has most likely resulted in a pattern change from former smoking habits to current NRT use. Therefore, even though some use NRT excessively suggesting a certain degree of habituation, the sustained use of NRT despite most wishing to quit strengthens the belief that the found association is actually based on dependence and not habituation.

### Long-term NRT use as a method for harm reduction?

Black et al. found that the presumptions among English smokers were that using NRT is addictive and harmful to health [23]. The findings in this study indicates that these beliefs persist, since 77% of the respondents felt addicted to NRT, and 40.9% of the respondents who stated that they wished to quit NRT feared it to be harmful to health. Only 12% of the respondents wished to continue using NRT, primarily because they enjoyed using the products and that it prevented possible relapse but also because they appreciated NRT being less hazardous than smoking. The scarcity of the respondents in this group cannot lead to any firm conclusions, but those not wishing to quit were mostly estimated to being low dependent, which could be connected to the lack of concern, as they may be more in control of their consumption than those estimated to being highly dependent on NRT.

There are currently no free counselling offers available in Denmark, for those long-term users wishing to quit NRT. The only current offer is an online service, where tapering out plans can be tailored according to the degree of consumption, both for smoking cessation and NRT use. This service is provided on behalf of the Danish Cancer Society, the Danish Pharmacies Association and The Danish Medicines and Health Agency (http://ekvit.dk/).

## Conclusion

The basic characteristics of the Danish long-term users match those found in previous studies on long-term users of NRT, but due to the study design, the generalizability of the population cannot be validated. A significant majority (88%) wished to quit NRT because of the cost of products, being tired of feeling addicted and fear of adverse health consequences. When applying the modified HSI, nicotine dependence on NRT was estimated primarily to be moderate to high. The strong association between TTFN and NRT use found gives reason to believe the validity of the modified HSI. Further studies are required for confirmation. A significant equivalence ratio of 0.86:1 was found when comparing current NRT use to recalled smoking, for those smoking 25 or less cig/day, indicating that nicotine dependence is still present, but new patterns of use concerning NRT may have replaced former smoking patterns. Better counselling of long-term users on the benefits of using NRT compared to smoking should be provided, for those who are chronically dependent, as well as support to quit long-term use of NRT if requested.

## Abbreviations

NRT: Nicotine replacement therapy
TTFN: Time to first NRT.
